# Description of surgical activity and mortality of oncological surgeries at the National Institute of Neoplastic Diseases (INEN) during the SARS-CoV-2 pandemic

**DOI:** 10.17843/rpmesp.2022.391.9772

**Published:** 2022-03-22

**Authors:** César Huaroto-Landeo, Kelly Kon-Liao, Glenda M. Falcon Pacheco, Ray Ticse

**Affiliations:** 1 Facultad de Medicina Alberto Hurtado, Universidad Peruana Cayetano Heredia, Lima, Peru. Universidad Peruana Cayetano Heredia Facultad de Medicina Alberto Hurtado Universidad Peruana Cayetano Heredia Lima Peru; 2 Instituto Regional de Enfermedades Neoplásicas del Sur, Arequipa, Peru. Instituto Regional de Enfermedades Neoplásicas del Sur Arequipa Peru; 3 Hospital Nacional Cayetano Heredia, Lima, Peru. Hospital Nacional Cayetano Heredia Lima Peru

*To the editor*. The Peruvian government declared a state of national emergency and mandatory social isolation on March 16, 2020, due to the SARS-CoV-2 pandemic ^1^. This restricted the activities that qualified as elective in all health care entities. Therefore, the aim of the study was to describe the surgical activity and mortality of oncologic surgeries at the National Institute of Neoplastic Diseases (INEN) during the SARS-CoV-2 pandemic in the years 2019-2020.

The INEN is a national referral center specialized in the diagnosis and treatment of neoplastic diseases, it is located in the eastern area of Lima in Peru. We described the number of scheduled and performed hospital oncologic surgeries by the Surgery Department of the INEN, between the months of March to December 2019 and compared them with the number of oncologic surgeries during the same period in 2020. We excluded patients who were treated in emergency areas, since including them would mean overestimating mortality rates. The Department of Surgery includes the surgical units of Abdomen, Head and Neck, Plastic and Reconstructive Surgery, Oncological Orthopedics, Gynecology, Neurosurgery, Breast and Soft Tissue, Thorax and Urology.

**Figure 1:**
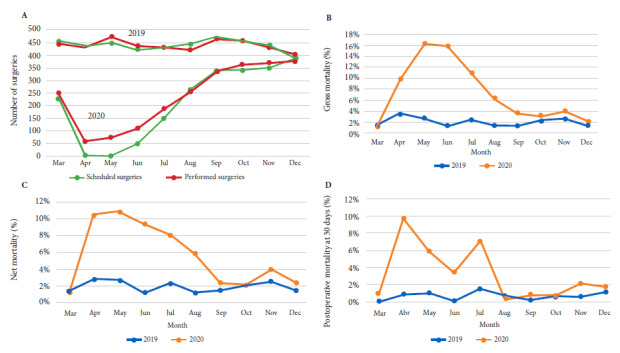
Quality indicators of the Surgery Department at the National Institute of Neoplastic Diseases (INEN) during the months of March to December 2019 and 2020. A. Scheduled and performed hospital surgeries. B. Percentage of gross mortality. C. Percentage of net mortality D. Percentage of postoperative mortality at 30 days.

Regarding hospital surgical activity, in 2019, the institution performed 5221 oncological surgeries, while in 2020 it performed 3268 surgeries, representing a decrease of 37% (Figure 1A) ^2^. The months of April and May 2020 stand out with four scheduled surgeries and 132 surgeries performed in total.

The lowest surgical productivity was observed at the beginning of the quarantine. We consider the following events to be involved: the beginning of confinement ^1^, the indefinite postponement of elective surgeries and the cancellation of outpatient consultations due to the reorganization of the Surgery Department to reduce the risk of contagion and detection of possible cases of COVID-19. Transportation restrictions also played a role, since INEN receives patients mostly from provinces far from Lima ^3^.

Data from 2020 show an increase in all mortality indicators (Figure 1B, 1C, 1D) (Supplementary Material) ^2^. There is higher gross mortality from April to December 2020, with May being the month of greatest difference with 16.4% in 2020 and 2.8% in 2019. Also, the maximum peak of 30-day postoperative mortality was observed in April 2020 with 9.8%.

The increase in mortality during 2020 can be explained by the decrease in surgical offer, the late access of oncology patients to health centers for fear of catching COVID-19 and by a system of teleconsultations with drawbacks. These changes in the health system delay timely diagnosis, lead to the presentation of pathologies in more advanced stages and increase emergency oncologic presentations ^4^. On the other hand, during the pandemic, the national program “Teleatiendo” ^5^ was promoted, which turned out to be insufficient, causing limitations in diagnosis and postoperative follow-up.

This analysis has limitations that should be acknowledged. First, we did not have access to some variables such as sex, age, place of origin, surgical risk, type and stage of cancer. Second, we did not have information on the specific postoperative cause of death. Third, it was not possible to inquire about the types of surgeries performed and their implication in the increase of mortality indicators during the year of the pandemic.

In conclusion, we found that surgical activity at the INEN was lower from March to December 2020 when compared to the equivalent period of the previous year. Similarly, all postoperative mortality indicators showed a substantial increase during the months of the health crisis. The delay of elective oncological surgeries brought cumulative consequences that led to deterioration of health, a worsening of quality of life and unnecessary deaths. It is advisable to implement a contingency plan for the surgical care of oncologic patients to ensure timely and quality care. In addition, it is essential to strengthen the telemedicine service in favor of comprehensive care for oncology patients.
