# Supplementary material for: Description of surgical activity and mortality of oncological surgeries at the National Institute of Neoplastic Diseases (INEN) during the SARS-CoV-2 pandemic
Source: Rev Peru Med Exp Salud Publica. 2022 Mar 22;39(1):120–1. doi: 10.17843/rpmesp.2022.391.9772 (PMC11397661; doi:10.17843/rpmesp.2022.391.9772)
Supplement: Supplementary material. — Available in the electronic version of the RPMESP. [file rpmesp-39-01-9772-s001.pdf]

MATERIAL SUPLEMENTARIO

Anexo 1. Definiciones operacionales de las figuras 1B, 1C y 1D.

Indicadores	Definiciones operacionales
Porcentaje de mortalidad bruta	Relación entre el número de fallecidos y el número de egresos (vivos y fallecidos) de todos los departamentos de cirugía del INEN. Se excluyeron las cirugías de emergencia.
Porcentaje de mortalidad neta	Relación entre el número de fallecidos que permanecieron más de 48 horas en el hospital y el número de egresos (vivos y fallecidos) que permanecieron más de 48 horas en el hospital de todos los departamentos de cirugía del INEN. Se excluyeron las cirugías de emergencia.
Porcentaje de mortalidad posoperatoria a los 30 días	Relación entre el número de fallecidos dentro de los 30 días posteriores a una cirugía y el número de cirugías realizadas de todos los departamentos de cirugía del INEN. Se excluyeron las cirugías de emergencia.

Rev Peru Med Exp Salud Publica
https://doi.org/10.17843/rpmesp.2022.391.9772
