# Supplementary material for: Description of surgical activity and mortality of oncological surgeries at the National Institute of Neoplastic Diseases (INEN) during the SARS-CoV-2 pandemic
Source: Rev Peru Med Exp Salud Publica. 2022 Mar 22;39(1):120–1. doi: 10.17843/rpmesp.2022.391.9772 (PMC11397661; doi:10.17843/rpmesp.2022.391.9772)
Supplement: Translation

# Translation: Descripción de la actividad quirúrgica y mortalidad de cirugías oncológicas en el Instituto Nacional de Enfermedades Neoplásicas (INEN) durante la pandemia por SARS-CoV-2

**Authors:** César Huaroto-Landeo, Kelly Kon-Liao, Glenda M. Falcon Pacheco, Ray Ticse

**Affiliations:** 1 Facultad de Medicina Alberto Hurtado, Universidad Peruana Cayetano Heredia, Lima, Peru.; 2 Instituto Regional de Enfermedades Neoplásicas del Sur, Arequipa, Peru.; 3 Hospital Nacional Cayetano Heredia, Lima, Perú.

Sr. Editor. En el Perú, debido a la pandemia por SARS-CoV-2, el gobierno declaró el 16 de marzo del año 2020 el estado de emergencia nacional y aislamiento social obligatorio ^1^. Con ello se restringieron las actividades que calificaban como electivas en toda entidad prestadora de salud. Por ende, el objetivo del estudio fue describir la actividad quirúrgica y mortalidad de cirugías oncológicas en el Instituto Nacional de Enfermedades Neoplásicas (INEN) durante la pandemia por SARS-CoV-2 en los años 2019-2020.

El INEN es un centro de referencia nacional en el diagnóstico y tratamiento de enfermedades neoplásicas, está ubicado en la zona este de Lima en Perú. Describimos el número de cirugías oncológicas hospitalarias programadas y realizadas por la Dirección de Cirugía del INEN, entre los meses de marzo a diciembre del 2019 y las comparamos con el número de cirugías oncológicas del 2020 del mismo periodo. Excluimos a los pacientes que se atendieron en áreas de emergencia, pues, incluirlos supone sobreestimar las tasas de mortalidad. La Dirección de Cirugía incluye los departamentos quirúrgicos de Abdomen, Cabeza y Cuello, Cirugía Plástica y Reconstructiva, Ortopedia Oncológica, Ginecología, Neurocirugía, Mamas y Tejidos Blandos, Tórax y Urología.

Sobre la actividad quirúrgica hospitalaria, en el 2019, la institución realizó 5221 cirugías oncológicas, mientras que en el 2020 realizó 3268 cirugías, lo que representa una disminución del 37% (Figura 1A) ^2^. Destacan los meses de abril y mayo del 2020 con cuatro operaciones programadas y 132 operaciones realizadas en total.

**Figura 1:** Indicadores de calidad de la Dirección de Cirugía en el Instituto Nacional de Enfermedades Neoplásicas (INEN) durante los meses de marzo a diciembre de los años 2019 y 2020. A. Operaciones hospitalarias programadas y realizadas. B. Porcentaje de mortalidad bruta. C. Porcentaje de mortalidad neta D. Porcentaje de mortalidad posoperatoria a los 30 días.

Los valores más bajos de productividad quirúrgica ocurrieron al inicio de la cuarentena. Consideramos como eventos implicados: el comienzo del confinamiento ^1^, la postergación indefinida de cirugías electivas y la cancelación de consultas externas en función a la reorganización de la Dirección de Cirugía para la reducción del riesgo de contagio y detección de posibles casos de COVID-19. También influyeron las restricciones en el transporte, debido a que el INEN recibe pacientes, en su mayoría, de provincias alejadas de Lima ^3^. 

Los datos referidos al 2020 muestran un incremento en todos los indicadores de mortalidad (Figura 1B, 1C, 1D) (Material suplementario) ^2^. Existe una mayor mortalidad bruta desde abril hasta diciembre del año 2020, siendo mayo el mes de mayor diferencia con 16,4% en 2020 y 2,8% en 2019. Asimismo, se observó un pico máximo de mortalidad posoperatoria a los 30 días en abril del 2020 con 9,8%. 

El aumento de la mortalidad durante el 2020 se puede explicar por el descenso de la oferta quirúrgica, el acceso tardío de los pacientes oncológicos a los centros de salud por temor a contagiarse de la COVID-19 y por un sistema de teleconsultas con inconvenientes. Estos cambios en el sistema de salud retrasan el diagnóstico oportuno, conllevan a la presentación de patologías en estadíos más avanzados y aumentan las presentaciones oncológicas de emergencia ^4^. Por otro lado, durante la pandemia se impulsó el programa nacional «Teleatiendo» ^5^, que resultó ser insuficiente, ocasionando limitaciones en los diagnósticos y en los seguimientos posoperatorios.

El presente análisis tiene limitaciones que deben ser reconocidas. Primero, no se tuvo acceso a algunas variables como el sexo, edad, lugar de procedencia, riesgo quirúrgico, tipo y estadio de cáncer. Segundo, no se tuvo información sobre la causa de muerte específica posoperatoria. Tercero, no se pudo indagar sobre los tipos de cirugías realizadas y su implicancia en el aumento de los indicadores de mortalidad, durante el año pandémico. 

En conclusión, hallamos que la actividad quirúrgica en el INEN, desde marzo a diciembre del 2020, fue inferior si se compara con el periodo equivalente del año anterior. De igual manera, todos los indicadores de mortalidad posoperatorios mostraron un incremento sustancial durante los meses de crisis sanitaria. El retraso de las cirugías electivas oncológicas trajo consecuencias acumulativas que provocaron el deterioro de la salud, un empeoramiento de la calidad de vida y muertes innecesarias. Es recomendable implementar un plan de contingencia para la atención quirúrgica de pacientes oncológicos que asegure una atención oportuna y de calidad. Además, es imprescindible fortalecer el servicio de telemedicina a favor de una atención integral del paciente oncológico.
